# Fungal Biodiversity in the Alpine Tarfala Valley

**DOI:** 10.3390/microorganisms3040612

**Published:** 2015-10-10

**Authors:** Claudia Coleine, Laura Selbmann, Stefano Ventura, Luigi Paolo D’Acqui, Silvano Onofri, Laura Zucconi

**Affiliations:** 1 Department of Ecological and Biological Sciences (DEB), Università degli Studi della Tuscia, Largo dell’Università, 01100 Viterbo, Italy; E-Mails: coleine@unitus.it (C.C.); onofri@unitus.it (S.O.); zucconi@unitus.it (L.Z.); 2 Institute of Ecosystem Study, National Research Council of Italy (CNR-ISE), I-50019 Sesto Fiorentino, Italy; E-Mails: stefano.ventura@ise.cnr.it (S.V.); dacqui@ise.cnr.it (L.P.D.)

**Keywords:** biological soil crusts, Denaturing Gradient Gel Electroforesis (DGGE), fungi, Internal Trascribet Spacers (ITS), Tarfala Valley

## Abstract

Biological soil crusts (BSCs) are distributed worldwide in all semiarid and arid lands, where they play a determinant role in element cycling and soil development. Although much work has concentrated on BSC microbial communities, free-living fungi have been hitherto largely overlooked. The aim of this study was to examine the fungal biodiversity, by cultural-dependent and cultural-independent approaches, in thirteen samples of Arctic BSCs collected at different sites in the Alpine Tarfala Valley, located on the slopes of Kebnekaise, the highest mountain in northern Scandinavia. Isolated fungi were identified by both microscopic observation and molecular approaches. Data revealed that the fungal assemblage composition was homogeneous among the BSCs analyzed, with low biodiversity and the presence of a few dominant species; the majority of fungi isolated belonged to the Ascomycota, and *Cryptococcus gilvescens* and *Pezoloma ericae* were the most frequently-recorded species. Ecological considerations for the species involved and the implication of our findings for future fungal research in BSCs are put forward.

## 1. Introduction

Biological soil crusts (BSCs) are key biotic components of terrestrial ecosystems composed of cyanobacteria, microalgae, fungi, lichens, mosses and heterotrophic bacteria in different proportions and their bio-products [1,2]; recently, archaeal populations have also been documented [3]. They occur in all hot, cold, cold-arid and semiarid regions of the world. Cyanobacteria filaments, microfungal hyphae, lichen rhizinae and the anchoring rhizoids of bryophytes aggregate together in the thin soil layer beneath the crust, promoting soil evolution and fertility and acting as protection from erosive forces [4,5,6]. The major importance of BSCs on primary succession in terrestrial ecosystems has only recently been recognized. Biological weathering of rock maintains a continuous supply of inorganic nutrients for plants in barren environments [7], and the microbes involved have an active role in element cycling, contributing to soil development and fertility, as well as plant establishment. Despite this, little attention has been paid to the mechanisms driving the first stages of colonization in a barren soil before plant colonization [8,9,10]. Even if soil crusts are extremely heterogeneous and develop under diverse climates, they are taxonomically and structurally very similar. Most of the frequently-recorded cyanobacteria, lichen and moss taxa have a cosmopolitan distribution, even at the species level. Fungi are one of most important components of BSCs, but their biodiversity and role remain poorly, if at all, investigated to date; few recent mycological studies were focused on BSCs in hot arid areas [11,12,13,14], while in Alpine and polar environments they were totally overlooked. The aim of this study was to examine fungal biodiversity in 13 samples of Arctic BSCs collected at different sites of Tarfala Valley (Sweden), a location mostly characterized by the high Arctic tundra, where environmental conditions are extreme and BSCs play a key role in creating a favorable habitat for plant establishment after ice melting.

## 2. Experimental Section

### 2.1. Study Area

**Figure 1:**
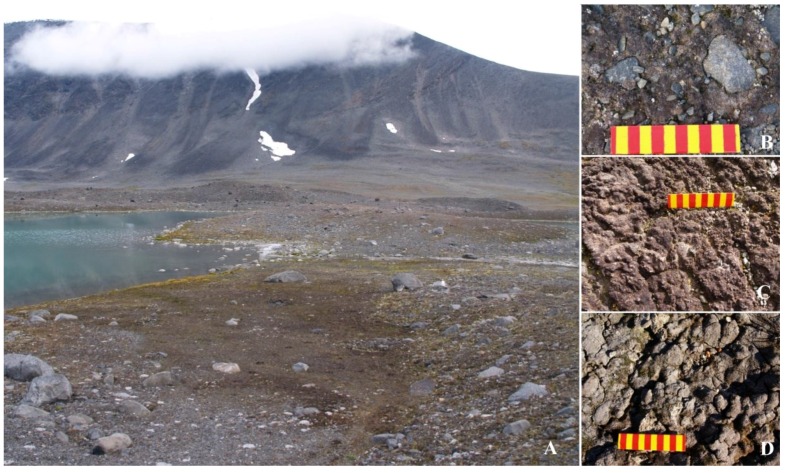
Tarfala Valley landscape (**A**); magnification of sites where C02 (**B**); C10 (**C**) and C12 (**D**) samples were collected. Scale: yellow/red bands 1 cm.

Tarfala Valley is a NW-SE-trending glacial trough with a drainage basin area of 20.6 km^2^ located on the eastern side of the Kebnekaise Massif in northern Sweden [15]. The trough floor has a minimum elevation of 800 m a.s.l., while peaks attain a maximum elevation of 2114 m a.s.l. on Kebnekaise, Sweden’s highest mountain. The Tarfalajåkk (Tarfala River) occupies the trough floor and flows southeast from Tarfalajaure Lake, which is situated at the head of the valley (Figure 1). A number of small cirque glaciers occur at higher elevations above 1400 m a.s.l. in the Tarfala area, and 3 valley glaciers, of which Storglaciären is the largest, descend to minimum elevations of approximately 1200 m a.s.l. on the western flank of the trough. The valley presents a variety of forms created by glacial, periglacial, fluvial, glaciofluvial, as well as mass movement processes. The mean annual temperature in the area is −3.3 °C, with minimum and maximum T around −20 °C and + 18 °C, respectively, while the mean annual precipitation is *ca*. 1000 mm. The vegetation is sparse and dominated by lichens, mosses and some ferns.

### 2.2. Sampling

Thirteen superficial soil crusts were collected from different sites of the moraines of Isfallglaciären and Kaskasatjåkkaglaciären glaciers and the east bank of Tarfalajaure Lake.

**Figure 2:**
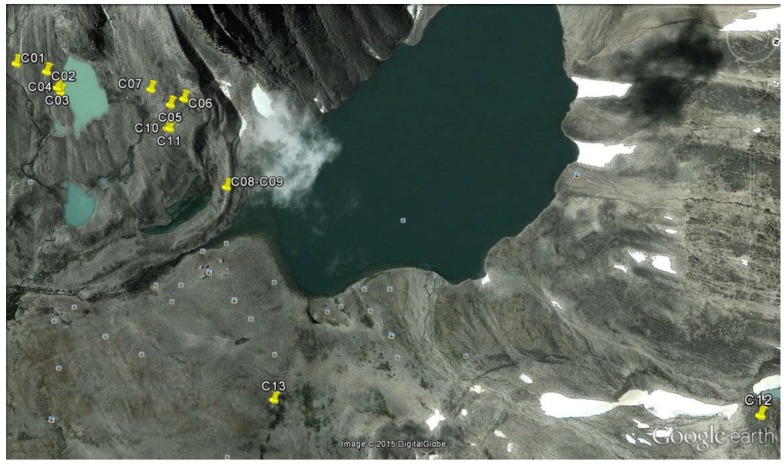
Map of the study area showing the location of the sampling sites.

Alpine Tarfala Valley (Figure 1 and Figure 2). Samples were collected with a sterile spatula along parallel transects by Stefano Ventura and Luigi Paolo D’Acqui in summer 2014 in an area ranging from 67°54′48.2″ N 18°35′19.8″ E to 67°55′16.3″ N 18°36′28.2″ E, comprising similar environments at comparable altitudes. Crust samples and collection data are listed in Table 1.

**Table 1:** List of the sampling sites.

Moraine Locations	Sample Number	Position	Altitude	Date of Sampling
Isfallglaciären	C01	67°54′48.2″ N 18°35′19.8″ E	1184 m	25 August 2014
Isfallglaciären	C02	67°54′50.8″ N 18°35′20.2″ E	1178 m	25 August 2014
Isfallglaciären	C03	67°54′51.8″ N 18°35′23.4″ E	1174 m	25 August 2014
Isfallglaciären	C04	67°54′52.1″ N 18°35′24.3″ E	1175 m	26 August 2014
Isfallglaciären	C05	67°55′02.1″ N 18°35′22.3″ E	1176 m	26 August 2014
Isfallglaciären	C06	67°55′03.3″ N 18°35′20.4″ E	1179 m	26 August 2014
Isfallglaciären	C07	67°55′00.1″ N 18°35′19.5″ E	1172 m	26 August 2014
Isfallglaciären	C08	67°55′08.2″ N 18°35′39.3″ E	1164 m	27 August 2014
Isfallglaciären	C09	67°55′08.2″ N 18°35′39.3″ E	1164 m	27 August 2014
Isfallglaciären	C10	67°55′02.1″ N 18°35′28.0″ E	1175 m	27 August 2014
Isfallglaciären	C11	67°55′02.3″ N 18°35′27.9″ E	1175 m	28 August 2014
Kaskasatjåkkaglaciären	C12	67°55′55.7″ N 18°36′06.4″ E	1439 m	30 August 2014
Tarfalajaure Lake	C13	67°55′16.3″ N 18°36′28.2″ E	1181 m	30 August 2014

### 2.3. Soil Analyses

Soil analyses were performed on samples <2 mm, sieved and dried at 40 °C. Total soil organic carbon was measured by an combustion elemental analyzer NA 1500 CHNS (Carlo Erba, Milan, Italy). Particle size distribution and pH in a 1:2.5 water suspension were determined according to the SISS (Società Italiana della Scienza del Suolo) methods [16].

### 2.4. Isolation

Crust samples were stored at −20 °C and processed within 3 months at the Systematic Botany and Mycology Laboratory of the University of Tuscia. Aliquots of 0.5 g of each crust were crushed, transferred to falcon tubes (50 mL) containing 49.5 mL Milli-Q sterile water and spread on MEA (malt extract agar; Applichem, GmbH, Darmstadt, Germany) in Petri dishes. Media were supplemented with chloramphenicol 100 ppm to prevent bacterial growth. Seeding was performed in triplicate; plates were incubated at 10 and 25 °C; growth was inspected weekly; and colonies were counted after 3 weeks. Colonies were picked and sub-cultured on fresh MEA.

For microscopy, mycelia from 15-day-old cultures were used. Slides were mounted in lactophenol and observed under a light microscope.

### 2.5. DNA Extraction, Amplification and Sequencing

Fungi were grown on agar slants (MEA) for 3 weeks. DNA extraction was carried out using the Nucleospin Plant kit (Macherey-Nagel, Düren, Germany), following the protocol optimized for fungi. PCR reactions were performed using BioMix (BioLine GmbH, Luckenwalde, Germany). Polymerase Chain Reaction (PCR) mixtures were prepared with 5 pmol of each primer and 40 ng of template DNA. Milli-Q sterile water was added at a final volume of 25 μL. Amplification was carried out using the MyCycler™ Thermal Cycler Bio-Rad Laboratories (GmbH, Munich, Germany). The ribosomal internal transcribed spacer region (ITS) was amplified and sequenced using ITS5 [17] and ITS4a [18] primers. Amplification was set as follows: 3 min at 95 °C for the first denaturation step, then 35 cycles of a denaturation step at 95 °C for 30 s, an annealing step at 55 °C for 30 s and an extension step at 72 °C for 30 s. The last extension was at 72 °C for 5 min. Products were purified using the Nucleospin Extract kit (Macherey-Nagel, Düren, Germany).

Sequencing reactions were performed according to the dideoxynucleotide method [19]. Fragments were analyzed by Macrogen Inc. (Seoul, Korea). Sequence assembly was done using the software ChromasPro v. 1.32 (Technelysium, Southport, Queensland, Australia).

### 2.6. Denaturing Gradient Gel Electrophoresis Fingerprinting Statistics, Quantification and Analysis

Total DNA was extracted from 1 g of fresh crust using a Nucleospin Plant Kit (Macherey-Nagel, Gmbh & Co. KG, Duren, Germany). For DGGE analysis, a semi-nested PCR was performed using primers with a (guanine cytosine) GC-clamp [20].

Fungal ITS rRNA genes were amplified from total DNA with primer sets ITS1F and ITS4 [21] in a semi-nested PCR performed in a total volume of 100 μL each with 4 μL of PCR products from the first amplification as the template (from PCR performed with primers ITS1F and ITS4). A GC-rich clamp (CGCCCGCCGCGCGCGGCGGGGGGGCGGGGGCC) was added to the 5ʹ end of ITS1F to improve subsequent band separation in DGGE [20]. The final amplified products were purified and/or concentrated using the Nucleospin Extract II Kit (Macherey-Nagel, Gmbh & Co. KG, Düren, Germany). A final DNA concentration of *ca*. 100 ng was loaded into each well for DGGE, and runs were performed on a DGGE-1 System (ELETTROFOR s.a.s., Scientific Instruments, Rovigo, Italy). The standard (Lane L1) was prepared on the base of the strains isolated from the samples studied.

Gel of 7.5% polyacrylamide (37.5:1 acrylamide:bisacrylamide) was run with 1× TAE (Tris-acetate EDTA) buffer at 200 V for 5 h. The optimum denaturing gradient for band separation was 20%–60% formamide and urea. Bands were visualized by staining for 40 min with GelRed (Biotiuminc, Hayward, CA, USA) solution (1.34 g NaCl, 66.7 pi GelRed and 200 mL dw). The gel images were analyzed with Phoretix 1D Pro software (CLIQS 1D Pro, Total Lab Ltd., Newcastle, UK). The dendrogram, relating band pattern similarities, was calculated with UPGMA (unweighted pair group method with arithmetic mean) algorithms.

Different diversity indexes were calculated: the richness index (*S*) indicates the number of bands detected in each lane; the Shannon diversity index (*H*ʹ) was calculated by assigning to each band a relative abundance as the ratio between the pixel intensity of the band and the sum of intensities detected in all bands in the same lane.

Portions of prominent bands were picked up, placed in 100 μL of elution buffer supplied with the Nucleospin Extract II Kit (Macherey-Nagel, Gmbh & Co. KG, Düren, Germany) and used for re-amplification with appropriate primers. PCR products were purified, sequenced and compared in the public domain using the BLASTN algorithm (http://www.ncbi.nlm.nih.gov/).

## 3. Results and Discussion

The BSCs samples were homogeneous in appearance; chemical analyses (Table 2) gave comparable pH values, very low organic nitrogen (not detectable) and carbon and a homogeneous texture in terms of size particles in all samples. The comparable low organic carbon content and no detectable nitrogen suggest a diffusely limited productivity [22] and a limited soil microbial activity and growth [23].

**Table 2:** Selected soil properties of the crust samples.

Samples	C (%)	Clay (%)	Silt (%)	Sand (%)	Skeleton (>2 mm) (%)	pH(H_2_O) 1:2.5	T (°C)
C01	0.09	2	41	57	18	6.1	6.5
C02	0.10	3	28	69	45	6.5	7.7
C03	0.09	3	30	66	9	6.5	7.4
C04	0.08	3	32	65	12	6.6	6.9
C05	0.17	2	15	84	37	6.6	13.8
C06	0.05	1	17	82	50	7.2	10.5
C07	0.18	1	14	85	10	7.2	13.4
C08	0.06	2	16	82	10	6.9	18.8
C09	1.21	0	30	69	49	6.4	4.1
C10	0.07	1	20	80	0	6.7	12.2
C11	0.04	3	23	74	48	6.8	10.9
C12	0.20	4	27	69	2	6.5	17.4
C13	3.22	2	51	47	26	6.3	20.0

Culture-dependent and culture-independent approaches are generally used as basic techniques to characterize microbial diversity. The first has the advantage of rendering the cultures available on which many studies can be done to elucidate the ecology and biology of the isolates; although plate counts are unlikely to reflect actual fungal abundance and diversity, therefore a culture independent approach is necessary; the combination of these approaches may give a clearer picture of the situation.

**Figure 3:**
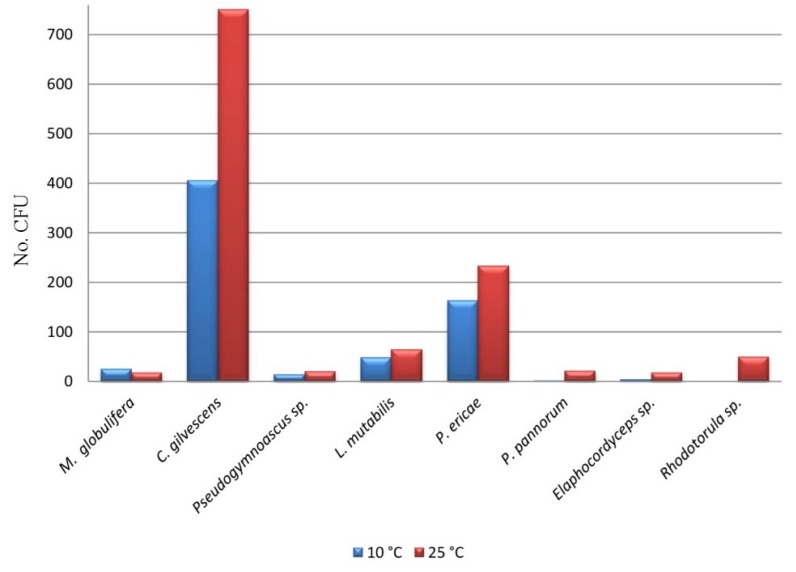
Abundance (number of CFU) of the isolated fungal strains after the 21st day at both isolation temperatures.

In this study, both culture-dependent and culture-independent approaches were used. Isolation, performed both at 10 and 25 °C, revealed the presence of only a few fungal taxa, most of which, identified by both morphological and molecular approaches, were represented by ascomycetous species and two basidiomycetous yeasts (Table 3). Isolation was successful at both of the temperatures used, but some species (*i.e*., *Pseudogymnoascus pannorum*, *Elaphocordyceps* sp. and *Rhodotorula* sp.) were detectable at 25 °C only. Moreover, overall, all of the species found grew best at 25 °C (Figure 3), suggesting that, most probably, none of them was psychrophile. Psychrotolerance is, as a rule, more recurrent in cold environments characterized by thermal fluctuations rather than permanent freezing [24,25].

Eight fungal taxa and 1905 CFU were obtained from the 13 BSC samples (Table 3).

The most frequent species isolated was *Cryptococcus gilvescens* (62.5%), while *P. pannorum* was the lowest (1.2%). The yeast genera *Cryptococcus* and *Rhodotorula* are commonly found in cold regions, including polar environments [26]. *C. gilvescens* is well known from cold environments; it was the most frequent species (51.2%) among the strains isolated from Alpine glaciers [27]; it was also recorded from sub-Antarctic [28] and Arctic sub-glacial habitats [29], including cryoconite holes in Svalbard, where it contributes to organic macromolecule degradation through cold-adapted enzyme secretion [30].

*Pezoloma ericae* was the second most common species with 20.9% frequency; it is related to ericaceous mycorrhizas (ERM) [31]. It was reported from boreal forests [32], where it forms ericoid mycorrhizas and colonizes the rhizoids of leafy liverworts. More information exists on ectomycorrhizal fungi associated with roots of Arctic plant species. *Ericaceae* are widespread in taiga and sub-Arctic tundra, where they are routinely colonized by ericoid mycorrhizas.

**Table 3:** Abundance (number of CFU) of fungal species isolated from the crust samples.

Taxa	C01	C02	C03	C04	C05	C06	C07	C08	C09	C10	C11	C12	C13	CFU (%)
*Mortierella globulifera* O. Rostr	31	-	-	-	-	-	-	-	-	21	-	-	-	2.7
*Cryptococcus gilvescens* Chernov & Babeva	250	23	139	43	90	-	228	8	12	-	264	134	-	62.5
*Pseudogymnoascus* sp.	-	7	4	-	-	-	-	-	22	-	-	-	-	1.7
*Lecythophora mutabilis* (J.F.H. Beyma) W. Gams & McGinnis	-	131	-	-	-	-	-	-	-	-	-	-	-	6.9
*Pezoloma ericae* (D.J. Read) Baral	15	-	59	3	30	25	-	11	-	140	42	10	63	20.9
*Pseudogymnoascus pannorum* (Link) Minnis & D.L. Lindner	-	-	-	-	1	21	-	-	-	-	-	-	-	1.2
*Elaphocordyceps* sp.	-	-	-	-	-	-	-	-	12	17	-	-	-	1.5
*Rhodotorula* sp.	33	-	-	-	-	-	13	-	3	-	-	-	-	2.6
CFU Total	329	161	202	46	121	46	241	19	49	178	306	144	63	1905

*P. pannorum* is also a fungus recurrent in cold regions, capable of growing at very low temperatures, down to −20 °C [33,34], and was frequently found in soil clone libraries from Interior Alaska [35]. *Lecythophora mutabilis*, even if present with a frequency of 6.9%, was isolated from sample C02 only. The remaining strains, present at a low percentage, were isolated from two or three samples only.

Both *P. ericae* and *C. gilvescens* were not only the most abundant, but also the most diffused species in the BSCs studied, being both present in 10 out of 13 samples. A similar situation, where there were few dominant species and widespread fungal types, with Ascomycota largely predominant, was observed in BSCs from hot arid areas in the Southwestern United States [36]. A similar structural diversity was reported also for BSC bacteria [37].

**Figure 4:**
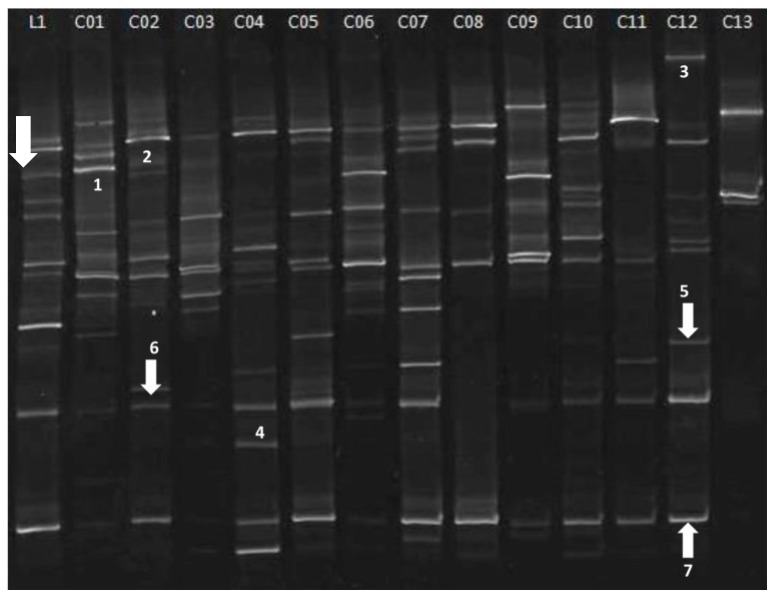
DGGE profiles of the fungal communities showing the comparison of the fungal soil communities in 13 different crust samples (C01–C13). L1: markers 1–7 correspond to bands present in different samples. White arrows: bands excised, re-amplified and sequenced.

The fingerprinting patterns shared a number of bands with the same electrophoretic mobility across different crust samples (Figure 4). As revealed by the software (picture not shown), band 1 was present in samples C01, C06 and C09; band 2 was present in samples C02-03-04-05-06-07-08-011; bands 5 and 7 were common to all samples; while band 6 appeared in all samples, except C07. Bands 3 and 4 were found in samples C12 and C04 only, respectively. The three dominant bands 5, 6 and 7 were excised, re-amplified and sequenced for comparison purposes. Sequencing results are presented in Table 4 with the identity % shared with sequences in the GenBank database. Bands 5 and 6 showed 99% identity with *C. gilvescens* and *P. ericae* respectively, band 7 100% with *Rhodotorula* sp., the first two also found as most the frequent and represented taxa in the cultivation test.

**Table 4:** Highest identities found for bands from the DGGE fingerprint.

Band ^a^	Highest match (NCBI Accession No.)	Identity (%) ^b^
5	*Rhodotorula* sp. (JF805370.1)	100%
6	*Cryptococcus gilvescens* (AB032678.1)	99%
7	*Pezoloma ericae* (AM887700.1)	99%

^a^ Bands are named according to Figure 4; ^b^ Identity represents the % similarity shared with sequences in the GenBank database; National Center for Biotechnology Information (NCBI).

The richness index, indicating the number of bands for each lane, ranged overall from 4 to 14 (Table 5), average 9.46 ± 2.84; these data are congruent with the ones concerning fungal assemblages in BSCs from major hot desert systems in the Southwestern United States [36]. The Shannon index varied between 0.57 and 1.06 (Table 5), average 0.9 ± 0.15; these results are much lower than what was found by Bates *et al*. in the BSCs of U.S. hot arid deserts [36] and indicate the presence of few dominant species.

**Table 5:** Richness index (*S*) and Shannon index (*H*ʹ) calculated with the software Phoretix 1D, based on the DGGE fungal community fingerprints of the 13 biological soil crust samples.

Sample	C01	C02	C03	C04	C05	C06	C07	C08	C09	C10	C11	C12	C13
Richness (*S*)	12	8	9	11	12	11	11	6	9	14	6	10	4
Shannon (*H*ʹ)	1.06	0.9	0.55	1.01	1.06	1.02	1.01	0.75	0.9	1.0	0.7	0.87	0.57

**Figure 5:**
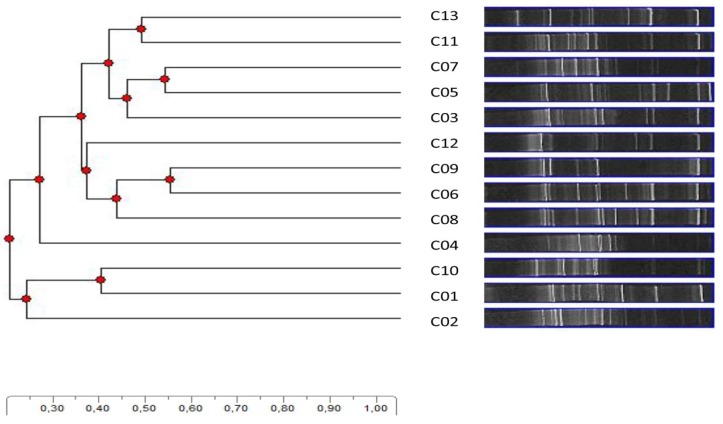
UWPGMA analysis of the DGGE profiles of the fungal communities.

The cluster analyses (Unweighted Pair Group Method with Arithmetic Mean, UWPGA; Figure 5) highlighted that the grouping obtained was not related to the sampling site, but rather intermixed: generally, samples collected far away from each other grouped together (see samples 11 and 13); similarly, samples from very close sites were separated (see samples 1–4). An ordinate clustering in fungal biodiversity DGGE profiles, related to the crust types compared, was observed in BSCs from hot arid environments [36]. Yet, in this study, the BSC samples compared were indeed quite homogeneous, both in appearance and chemical composition, and, definitely, in biodiversity.

## 4. Conclusions

This is the first study dealing with the biodiversity characterization of fungal assemblages in Arctic BSC samples; data revealed that all samples analyzed were characterized by extremely low CFU numbers and biodiversity, with the presence of two dominant species, *C. gilvescens* and *P. ericae*, and occasional and poorly representing a few others. These two dominant taxa were detected by both culture-dependent and -independent approaches. Even the biodiversity variation among the samples was low, regardless of the sampling site; this was probably related to the very similar composition and appearance of soil beneath BSCs. The predominance of the two species is probably related to their ecology. *C. gilvescens* is recurrent in Alpine and polar environments, suggesting that it easily adapts in cold conditions. *P. ericae* is the host of *Calluna vulgaris*, a plant that frequently occurs in moorland of North Europe, including Sweden. Besides, the presence of a typical mycorrhizal fungus in the BSCs studied may represent the first support to the ‘fungal loop’ hypothesis, which suggests a key role for fungi in mediating nutrient exchange between BSCs and patches of vegetation in arid landscapes [38]. The number of fungal species found in these works was lower rather than what was reported for BSCs in hot-arid locations [36], but it is difficult at this stage to sort out if this is related to the cold itself or to the sampling area. Future studies on additional samples from cold environments may allow making more reliable and supported hypotheses. A similar pattern with a few dominant types and a patchy distribution of additional phylotypes had been observed for both bacteria and fungi from BSCs in hot-arid locations [36,37], but the fungal species reported in the literature were different from what was found in the present study, with a large predominance of darkly-pigmented taxa from the Pleosporales [13].

Additional studies are needed from other sites, a larger geographic range and different cold locations to have a clearer picture of the diversity and the relationship between the ecology of species and geography and to clarify if a BSC-exclusive or BSC-specific fungal flora exists. With this aim, further samples from both Arctic and Antarctic environments will be analyzed in the near future.

The extreme environmental conditions of the Arctic and, even more, Continental Antarctica allow a limited period of metabolic activity. A deeper understanding of the structure and composition of BSCs in polar ecosystems is the key to the protection of these fragile environments and to predict how ongoing climate change would affect their structure, composition and distribution.
